# Enhanced anti-influenza virus activity of saliva following toothbrushing

**DOI:** 10.1038/s41405-025-00355-3

**Published:** 2025-07-19

**Authors:** Yusuke Kubo, Taku Iwamoto, Seiichi Tobe, Riho Tateyama-Makino, Kota Tsutsumi, Keiichi Tsukinoki, Kei Kurita

**Affiliations:** 1 Research & Development Headquarters, Lion Corporation, Edogawa-ku, Tokyo, Japan; 2 Department of Environmental Pathology, Kanagawa Dental University, Yokosuka, Kanagawa Japan

**Keywords:** Oral hygiene, Infection control in dentistry

## Abstract

**Objective:**

Influenza, a respiratory infection caused by the influenza virus, has been associated with good oral hygiene, which correlates with a reduced incidence of the disease. Saliva possesses inherent antiviral properties against the influenza virus. However, the relationship between toothbrushing, a common oral hygiene practice, and the antiviral activity of saliva remains poorly understood. This study aimed to evaluate the effect of toothbrushing on the anti-influenza virus activity of saliva.

**Materials and methods:**

Sixteen adults without oral disease participated in this open-label, single-arm study. Resting saliva and mouth-rinsed water samples were collected before toothbrushing. Participants then brushed their teeth with a toothbrush and toothpaste for five minutes, after which additional saliva and mouth-rinsed water samples were collected at five minutes and one-hour post-brushing. The total bacterial amount in the mouth-rinsed water was measured by qPCR. The anti-influenza virus activity of saliva was determined using the TCID₅₀ method.

**Results:**

Saliva’s anti-influenza virus activity increased significantly five minutes after toothbrushing compared to pre-brushing levels, but no significant difference was observed at 1 h, as follows [Δlog, median (min-max)]: Before brushing: 0.625 (−0.25–1.75), at 5 min: 1.25 (0.5–2), and at 1 h: 0.75 (0.5–2). A correlation analysis between total bacterial amount and antiviral activity revealed a negative correlation.

**Conclusions:**

Improving the oral environment through toothbrushing enhances salivary antiviral activity. Maintaining oral hygiene may help prevent influenza virus infection.

## Introduction

Influenza, a respiratory infection caused by the influenza virus, contributes significantly to global morbidity annually [1]. Each year, seasonal influenza impacts nearly one billion individuals, with 3 to 5 million cases advancing to severe illness [1]. These viruses primarily infect epithelial cells in the upper respiratory tract via the oral and nasal cavities [2]. The oral cavity, as a key entry point for influenza viruses, is lined with mucosal surfaces that are continuously bathed in saliva [3]. Saliva plays a critical role in defending the oral mucosa by providing both a physical barrier and immunological protection [3]. Components of saliva, including secretory IgA (sIgA), scavenger receptor cysteine-rich glycoprotein 340 (gp340), histatins, human neutrophil defensins (HNPs), and lysozyme, exhibit potent antibacterial and antiviral properties [4–11]. These salivary components are responsible for the natural immunity of the oral cavity [3, 12]. As such, saliva is essential in mitigating influenza virus infection.

Oral hygiene practices, such as toothbrushing, are crucial for maintaining and improving the oral environment and preventing oral diseases like dental caries and periodontal disease. A retrospective observational cohort study demonstrated that good oral hygiene correlates with a reduced incidence of influenza [13]. Furthermore, an interventional study reported that older adults using daycare services who received weekly professional oral care from dental hygienists experienced a lower incidence of influenza compared to those who did not receive such care [14]. These findings indicate that maintaining and improving oral hygiene can serve as an effective strategy for preventing influenza.

Based on these observations, we hypothesized that improving the oral environment through oral hygiene practices could enhance the anti-influenza virus activity of saliva. However, the specific impact of toothbrushing with toothpaste, one of the most common oral hygiene behaviors, on the antiviral activity of saliva remains unclear. To address this issue, the present study evaluated the anti-influenza virus activity of saliva collected before and after toothbrushing to determine its influence on enhancing salivary antiviral properties.

## Materials and methods

### Study design

This study adheres to the Ethical Guidelines for Medical and Health Research Involving Human Subjects (Japanese government regulations, revised March 10, 2022) and the Declaration of Helsinki (2013 revision). Additionally, the study was registered with the University Hospital Medical Information Network Clinical Trials Registry (UMIN-CTR: UMIN000054504), meeting ICMJE standards. The Lion Corporation Clinical Trial Review Committee approved the study on February 7, 2023 (authorization number: 374). Prior to participating in the study, all participants provided written informed consent.

The study was conducted between 9:00 and 10:30 a.m. Participants were instructed to refrain from eating, drinking (except 200 mL of water), and performing any oral hygiene from 11:00 p.m. the night before testing until 9:00 a.m. on the test day. Saliva samples were collected as follows: pre-brushing samples: resting saliva was collected by having participants spit into a 25 mL plastic tube for three minutes. Participants then rinsed their mouths with 3 mL of distilled water for 10 s and spat the discharge into a separate 25 mL plastic tube; post-brushing samples: participants brushed their teeth for five minutes using a commercially available toothbrush (Clinica Advantage 4 Rows Compact Regular, Lion Corporation) and toothpaste (Systema EX Toothpaste Medical Cool, Lion Corporation). The participants then rinsed their mouths with 10 mL of water after brushing. Once five minutes had elapsed, resting saliva (collected as before) and mouth-rinsed water (collected by vigorously rinsing the mouth with 3 mL of sterile water for 10 s) were sampled; one-hour post-brushing samples: resting saliva and mouth-rinsed water were collected again one hour after brushing. At this point, participants were instructed to refrain from consuming any food or drink.

### Participants

The sample size was determined using the EZR (Easy R, Saitama Medical Center, Jichi Medical University, Saitama, Japan) package on R (ver.4.0.2) based on an effect size of 0.173, an α level of < 0.05, and 80% power. The result indicated that the required sample size was 15. Assuming that some participants might not fulfill the inclusion criteria or would meet the exclusion criteria, 26 candidates initially provided written informed consent. The selected candidates underwent a dental examination conducted by a dentist to assess decayed, missing, and filled teeth (DMFT) and to evaluate periodontal health status using probing pocket depth (PPD) and bleeding on probing (BOP). Resting saliva samples were also collected during the screening process. Participants diagnosed with a carious cavity, a PPD of 4 mm or larger, or BOP were excluded. Finally, 16 participants met the selection criteria, were not excluded based on the exclusion criteria, and were enrolled in the study (Supplementary File 1). These participants were further surveyed regarding their influenza vaccination history.

### Measurement of anti-influenza virus activity

The anti-influenza virus activity of saliva was assessed using the Median Tissue Culture Infectious Dose (TCID₅₀) method. The test employed the Influenza A virus (H1N1) strain A/PR/8/34 ATCC VR-1469 and canine kidney tubular epithelial cells (MDCK cells) as host cells. Procedures for MDCK cell culture, virus preparation, and infectivity evaluation followed JIS L 1922 guidelines [15]. Cell Culture: MDCK cells were cultured in Eagle’s Minimal Essential Medium (EMEM, Catalog no. M4655-500ML, Sigma-Aldrich) supplemented with 1% penicillin-streptomycin (Catalog no. P4333-100ML, Sigma-Aldrich) and 10% fetal bovine serum (Catalog no. 173012, Sigma-Aldrich) under a 5% CO₂ atmosphere at 37 °C. Saliva samples were incubated with the influenza virus to measure antiviral activity. A 10 µL solution of influenza virus (average initial titer: 6.8 × 10⁷ TCID₅₀/mL) was mixed with 90 µL of saliva and incubated at 37 °C for 60 min. The mixture was serially diluted ten-fold four times with EMEM, added to MDCK cells in a 96-well microplate, and incubated at 34 °C for 60 min. After washing, EMEM containing 1.5 ppm trypsin was added, and the plate was incubated at 34 °C for four days. Viral infectivity was assessed by counting wells showing cell degeneration via optical microscopy. The infectivity titer was expressed as TCID₅₀, and the antiviral activity of saliva was expressed as the log₁₀ reduction rate (Δlog) using the formula:

1 $$\varDelta \log =\log ({Infectivity\; titer\; with\; control})-\log ({Infectivity\; titer\; with\; saliva})$$

A Δlog value of 1 indicates a 90% reduction in viral infectivity.

### Quantitative real-time PCR

According to the report of Yama, et al., Quantitative real-time PCR was performed on extracted genomic DNA to quantify total bacterial amount in the mouth-rinsed water using SsoAdvanced Universal Probes Supermix (Bio-Rad Laboratories Inc., Hercules, CA, USA) on a CFX Duet system (Bio-Rad) [16].

### Statistical analysis

All statistical analyses were performed using EZR (Easy R, Saitama Medical Center, Jichi Medical University, Saitama, Japan), a statistical package based on R (version 4.0.2). Paired comparisons were conducted using the Friedman test followed by the Bonferroni-corrected Wilcoxon signed-rank test, with the significance level adjusted to 0.05/3 = 0.0167 for three pairwise comparisons within groups. A *P* value of < 0.05 was considered statistically significant. Finally, a correlation analysis was performed using the Spearman rank correlation coefficient.

## Results

### Saliva anti-influenza virus activity

**Fig. 1:**
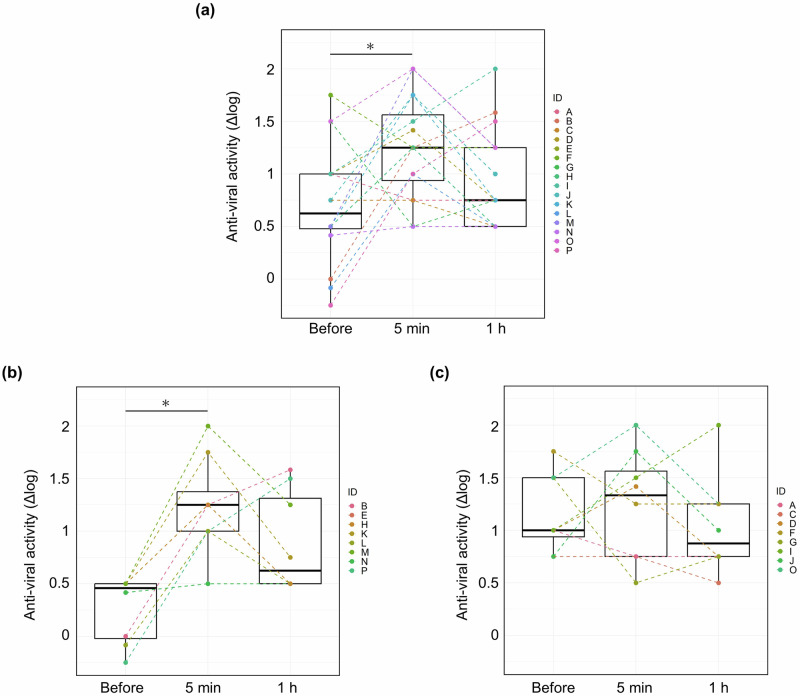
Salivary anti-influenza virus activity. The anti-influenza virus activity of saliva was measured at three time-points: before, 5 min after, and 1 h after toothbrushing. The horizontal axis represents the saliva collection time, and the vertical axis indicates anti-influenza virus activity. **a** Analysis of saliva from all participants (*n* = 16). **b** Analysis of saliva from participants with low antiviral activity before toothbrushing (*n* = 8). **c** Analysis of saliva from participants with high antiviral activity before toothbrushing (*n* = 8). The Wilcoxon signed-rank test, corrected using the Bonferroni method, was used for statistical analysis. The significance threshold was set at 0.0167 (0.05/3). Different letters **a, b** denote statistically significant differences (*P* < 0.0167).

Subject characteristics are summarized in Table 1. Anti-influenza virus activity was assessed using saliva samples collected at three time points: before brushing, 5 min after brushing, and 1 h after brushing. The findings revealed a significant increase in salivary anti-influenza virus activity 5 min after toothbrushing compared to pre-brushing levels (Fig. 1a). To evaluate the impact of baseline anti-influenza virus activity on the post-brushing response, a stratified analysis was conducted. Participants were divided into two groups based on their pre-brushing anti-influenza virus activity: a low-activity group (*n* = 8) and a high-activity group (*n* = 8). In the low-activity group, the Δlog value ranged from −0.25 to 0.5, with a significant increase in salivary anti-influenza virus activity observed 5 min after brushing (Fig. 1b). Conversely, in the high-activity group, the Δlog value ranged from −0.75 to 1.75, and no significant changes in activity were observed before and after toothbrushing (Fig. 1c).

**Table 1:** Background information on subjects.

*n* [Men / Women]	16 [7 / 9]
Age (years, mean ± SD)	31.6 ± 7.4
Total number of teeth	28.3 ± 1.7
Number of DMFT	5.8 ± 5.0
Number of filled teeth	5.1 ± 4.3
Number of missing teeth	0.6 ± 1.4
Maximum PPD (mm)	3.3 ± 0.6
Mean PPD (mm)	2.3 ± 0.1
Number of teeth with PPD ≥ 4 mm	0.6 ± 1.3
Number of teeth with BOP	1.4 ± 1.7
Number of participants who received influenza vaccinations (self-reported, Fall 2022 onwards)	15

Values are presented as mean ± SD (*n* = 16). *DMFT* decayed, missing, and filled teeth, *PPD* probing pocket depth, *BOP* bleeding on probing.

### Relationship between salivary anti-influenza virus activity and total bacterial amount in the mouth-rinsed water

To explore the relationship between oral cleanliness and salivary anti-influenza virus activity, total bacterial amount was measured in mouth-rinsed water samples collected prior to saliva collection. An additional figure file presents the total bacterial amount results (Supplementary File 2). To eliminate individual differences in pre-brushing values and to standardize across participants, we calculated the difference (Δ value) by subtracting the total bacterial load and antiviral activity values at 5 min and 1 h after toothbrushing from their respective pre-brushing values. This allowed us to determine the changes in these parameters attributable to toothbrushing. The correlation analysis revealed a negative correlation between the Δ value of total bacterial load and the Δ value of antiviral activity at 5 min (correlation coefficient = −0.539) and at 1 h (correlation coefficient = −0.463).

## Discussion

This study demonstrates that toothbrushing enhances the oral environment and significantly boosts the anti-influenza virus activity of saliva. To our knowledge, this is the first report showing that oral hygiene practices, specifically toothbrushing, can improve salivary antiviral activity against influenza.

The anti-influenza virus activity of saliva increased notably after toothbrushing (Fig. 1a). Before brushing, a wide individual variation in salivary antiviral activity (Δlog) was observed, ranging from −0.25 for the lowest subject to 1.75 for the highest (Fig. 1a). Similar individual differences have been reported by Kobayashi et al., who found that salivary anti-influenza activity varied significantly across individuals, with minimum activity as low as 2% and maximum activity reaching 97% [17]. In light of these variations, a stratified analysis was conducted by grouping subjects based on their pre-brushing salivary antiviral activity. The analysis revealed that individuals with lower initial antiviral activity experienced a significant enhancement 5 min after brushing, with a median increase of 6.2 times (Fig. 1b). In contrast, individuals with higher initial antiviral activity maintained higher values after brushing, with no significant change. This finding suggests that toothbrushing may be particularly beneficial for individuals with low baseline salivary anti-influenza activity.

Some studies have reported that surfactants in toothpaste possess the ability to directly inactivate influenza viruses [18, 19]. The SDS contained in the toothpaste used in this study has been reported to exhibit in vitro antiviral activity at concentrations of 0.1% or higher [20]. While the concentration of SDS in the commercial toothpaste used in this study is unknown, typical toothpastes contain 0.5–2% SDS [21]. Therefore, upon evaluating the in vitro antiviral activity (⊿LOG) of several commercial toothpastes containing SDS, all toothpastes demonstrated an antiviral activity of ⊿LOG > 2 (Supplementary file 3). This suggests that toothpastes containing SDS may have in vitro antiviral activity. On the other hand, during regular toothbrushing, toothpaste is diluted by saliva and rinsing after brushing. Toothpaste is typically diluted three to four times during brushing—a dilution ratio commonly used in in vitro evaluations of toothpaste components [22–24]. Moreover, after brushing, assuming that rinsing with 10 mL of water results in a 20-fold dilution and saliva flow of 2.5 mL over 5 min results in a five-fold dilution, the concentration of SDS in saliva 5 min after brushing is estimated to be less than 0.01% (optimistically estimated at 0.008%). Therefore, although the toothpaste itself has antiviral activity, it is considered that the chemical effects of the toothpaste have a minimal impact on the antiviral activity of saliva collected 5 min after brushing. This study did not include a brushing group without toothpaste. Therefore, to investigate the mechanism of enhanced antiviral activity due to brushing, future studies should include tests that separate the mechanical effects of brushing from the chemical effects of toothpaste.

**Fig. 2:**
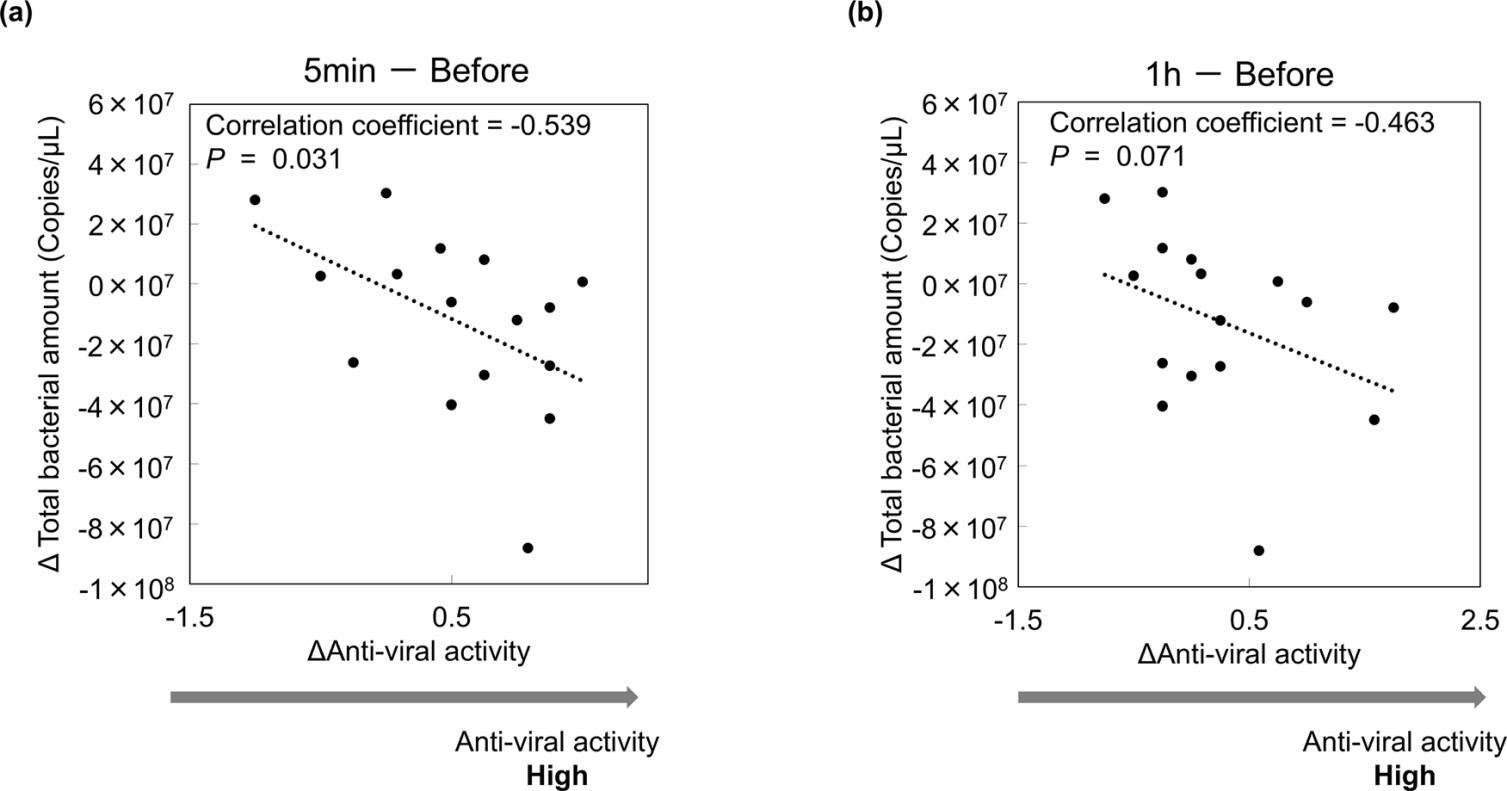
Relationship between salivary anti-influenza virus activity and total bacterial amount in mouth-rinsed water. The anti-influenza virus activity of saliva and total bacterial amount in mouth-rinsed water were measured at three time points: before, 5 min after, and 1 h after toothbrushing. The horizontal axis represents anti-influenza virus activity, while the vertical axis indicates total bacterial amount. To eliminate individual differences in pre-brushing values and for standardization among participants, we calculated the difference (Δ value) by subtracting the values of total bacterial load and antiviral activity at 5 min and 1 h after toothbrushing from the values before toothbrushing. Each plot represents individual sample values. **a** Correlation analysis of Δvalue 5 min after brushing. **b** Correlation analysis of Δvalue 1 h after brushing. Dotted lines represent regression lines. Statistical analysis was performed using Spearman rank correlation coefficients.

To explore whether the improved oral environment contributed to the observed increase in salivary antiviral activity, a correlation analysis was conducted between salivary anti-influenza activity and the total bacterial amount in mouth-rinsed water, an indicator of oral cleanliness, a factor of oral hygiene.(16–19) A negative correlation was found between the Δ value of total bacterial load and the Δ value of antiviral activity at 5 min (correlation coefficient = −0.539) and at 1 h (correlation coefficient = −0.463)(Fig. 2). These results suggest that the reduction of oral bacteria and improvement of oral cleanliness due to toothbrushing are related to the enhancement of antiviral activity in saliva through tooth brushing. In addition, saliva contains components with antibacterial and antiviral effects. Previous studies have demonstrated that various salivary components, including sIgA, scavenger receptor cysteine-rich glycoprotein 340 (gp340), histatins, human neutrophil defensins (HNPs), and lysozyme, exhibit antiviral activity against influenza A viruses [4–10]. Of these, particularly sIgA, gp340, HNP, and lysozyme also possess antimicrobial properties, indicating they have both antibacterial and antiviral effects [4]. Therefore, it is suggested that the improvement in anti-influenza virus activity of saliva observed after tooth brushing is associated with a reduction in oral bacterial load. In other words, when the level of oral bacteria is high, which presumably interact with the antiviral components in saliva and nullify the action of the components in saliva against the influenza virus. However, the underlying mechanism and the extent of this occurrence will be examined in future studies.

This study has some limitations. As this study is an open-label single-arm trial, there is a risk of inherent bias, and factors other than toothbrushing, such as recruiting only participants with good oral condition, and the average age (mean ± SD) being 31.6 ± 7.4 (skewing younger), may have influenced the results. Since the experiments were conducted in vitro to quantify influenza virus infection in cultured cells, the direct implications and preventive effects of toothbrushing on influenza infection in humans were not conveyed. Further research is necessary to investigate the impact of toothbrushing on actual influenza infection rates. However, previous research has established a connection between oral hygiene practices and a reduced risk of influenza infection, including studies showing that professional oral care by dental hygienists decreases the likelihood of influenza infection [12, 13]. These findings support the conclusion that toothbrushing contributes to the inhibitory effects of saliva on influenza viral infection by improving oral cleanliness.

## Conclusion

This study demonstrates that toothbrushing enhances the anti-influenza virus activity of saliva significantly in vitro. It suggests the importance of maintaining and improving oral health through regular oral hygiene practices, which may support saliva’s antiviral properties. These findings suggest that promoting a healthy oral environment through habits like toothbrushing can serve as an effective strategy for preventing influenza virus infections.

## Supplementary information

Inclusion Criteria and Exclusion Criteria

Total bacterial amount in mouth-rinsed water (Copies/μL)

Measurement of Antiviral Activity of Commercial Toothpastes

## Data Availability

The data presented in this study are available upon request from the corresponding author.
