# Supplementary material for: Enhanced anti-influenza virus activity of saliva following toothbrushing
Source: BDJ Open. 2025 Jul 19;11:68. doi: 10.1038/s41405-025-00355-3 (PMC12276237; doi:10.1038/s41405-025-00355-3)
Supplement: Supplementary file 1 — Inclusion Criteria and Exclusion Criteria [file 41405_2025_355_MOESM1_ESM.docx]

Supplementary file 1

Inclusion Criteria

- Men and women aged 20 to 59.
- Brushing at least twice daily with a toothbrush and toothpaste.
- Brushing after dinner and before bedtime.
- At least 20 remaining teeth.
- Ability to collect at least 600 µL of saliva by spitting for 3 min during the screening evaluation.
- Individuals who have provided written, voluntary consent to participate in the study.

Exclusion Criteria

- Planning to undergo dental treatment (including preventive visits) during the evaluation period.
- Planning to participate in oral care-related examinations (e.g., toothbrush, toothpaste, mouthwash, dental floss) during the evaluation period.
- Undergoing orthodontic treatment.
- Using bridges, dentures, or implants.
- Diagnosed with a carious cavity
- Bleeding on probing (BOP) from probing pocket depth (PPD) ≥ 4 mm.
- Having taken antibiotics within the past 3 months.
- Smoking.
- Regularly consumes alcohol (more than 5 days a week), exceeding 40 g/day for men and 20 g/day for women.
- Diagnosed by a physician as having blood diseases (e.g., aplastic anemia), infectious diseases (e.g., chickenpox, cytomegalovirus infection, measles, infectious mononucleosis), diabetes, renal disease, liver disease, chronic gastritis, colitis, systemic lupus erythematosus, cancer, and immune deficiency disease, alcoholism, or undernourished.
- Currently pregnant or potentially pregnant.
- Any other individuals deemed unsuitable for the study by the principal investigator
