# Supplementary material for: Enhanced anti-influenza virus activity of saliva following toothbrushing
Source: BDJ Open. 2025 Jul 19;11:68. doi: 10.1038/s41405-025-00355-3 (PMC12276237; doi:10.1038/s41405-025-00355-3)
Supplement: Supplementary file 2 — Total bacterial amount in mouth-rinsed water (Copies/μL) [file 41405_2025_355_MOESM2_ESM.docx]

Supplementary file 2

Total bacterial amount in mouth-rinsed water (Copies/μL)

**Results**

Total bacterial amount (Copies/μL) were measured in mouth-rinsed water collected at three time points: before, 5 min after, and 1 h after toothbrushing as follows [median(min-max)]: Before: 6.5x10^7^(1.5x10^7^-1.8x10^8^), 5min: 3.9x10^7^(7.5x10^6^-1.3x10^8^), 1h: 3.4x10^7^(8.0x10^6^-7.7x10^7^).
