# Supplementary material for: Enhanced anti-influenza virus activity of saliva following toothbrushing
Source: BDJ Open. 2025 Jul 19;11:68. doi: 10.1038/s41405-025-00355-3 (PMC12276237; doi:10.1038/s41405-025-00355-3)
Supplement: Supplementary file 3 — Measurement of Antiviral Activity of Commercial Toothpastes [file 41405_2025_355_MOESM3_ESM.docx]

Supplementary file 3. Measurement of Antiviral Activity of Commercial Toothpastes

Material and method

・Sample

Seven types of commercially available toothpastes, labeled A-G, were used.

・The anti-influenza virus activity

The anti-influenza virus activity was measured according to the section "Measurement of Anti-Influenza Virus Activity" using the Median Tissue Culture Infectious Dose (TCID₅₀) method. Toothpaste samples were incubated with the influenza virus to measure antiviral activity. A 0.1 mL solution of influenza virus was mixed with 0.9g of the toothpaste sample and incubated at 37°C for 3 minutes. Subsequently, 10mL of SCDLPB was added and stirred. The mixture was serially diluted 10-fold four times with EMEM, added to MDCK cells in a 96-well microplate, and the plate was incubated at 34°C for six days.

Results

The antiviral activity (⊿LOG) of each sample was as follows:

Toothpaste A：⊿LOG >4

Toothpaste B：⊿LOG >4

Toothpaste C：⊿LOG 2.3

Toothpaste D：⊿LOG 2.4

Toothpaste E：⊿LOG >4

Toothpaste F：⊿LOG >3

Toothpaste G：⊿LOG >3
